# Effects of Combination Taping Technique on Disability, Functional Capacity, and Knee Isokinetic Torque in Patients with Knee Osteoarthritis: A Blinded Randomized Controlled Study

**DOI:** 10.3390/healthcare12242542

**Published:** 2024-12-16

**Authors:** Nouf H. Alkhamees, Osama R. Abdelraouf, Olfat Ibrahim Ali, Zizi M. Ibrahim, Amal A. Elborady

**Affiliations:** 1 Department of Rehabilitation Sciences, College of Health and Rehabilitation Sciences, Princess Nourah bint Abdulrahman University, Riyadh 11671, Saudi Arabia; nhalkhamees@pnu.edu.sa; 2 Physical Therapy Program, Batterjee Medical College, Jeddah 21442, Saudi Arabia; pt4.jed@bmc.edu.sa (O.R.A.); olfat.sayed@bmc.edu.sa (O.I.A.); 3 Department of Biomechanics, Faculty of Physical Therapy, Cairo University, Giza 12613, Egypt; dr_mouly@cu.edu.eg

**Keywords:** osteoarthritis, knee taping, physiotherapy, disability, isokinetic torque, aerobic capacity

## Abstract

Background: Treatments that combine both elastic and rigid taping in knee osteoarthritis have not yet been investigated in the literature. Thus, the purpose of the present study is to investigate how the combination taping technique affects functional status, disability, and quadricep isokinetic torque in cases of knee osteoarthritis. Patient Methods: A total of fifty-four patients were assigned to the experimental group or control group. Conventional physical therapy was provided to both groups, in addition, participants in the experimental group also received combination taping. Disability, functional status, and isokinetic quadriceps torque were assessed at baseline, six weeks (post-intervention), and twelve weeks (follow-up). Results: MANOVA showed that post-intervention measurements were significantly better than baseline measurements of both groups, except for isokinetic quadriceps torque, which showed a nonsignificant difference in the control group. The control group’s follow-up measurements revealed nonsignificant differences from those taken after the intervention, whereas the experimental group’s differences were significant excluding isokinetic quadriceps torque. Measurements taken at post-intervention and follow-up revealed that the experimental group had significantly improved compared to the control groups. Conclusions: Combination taping was found to be more beneficial when used in addition to conventional physical therapy than when used alone in knee OA.

## 1. Introduction

Osteoarthritis (OA) is the most prevalent joint disease currently, which affects nearly 34% of those aged 65 and older [1,2]. According to the latest report published by the World Health Organization, there has been a significant increase of 113% for people living with osteoarthritis compared to data from 1990 [3]. Many factors could contribute to developing OA, such as joint injury due to obesity, aging, or possible hereditary influences [4]. As osteoarthritis is regarded as being one of the main causes of deformity in general, and on the knee joint in particular, this in turn results in large medical expenses and a poor quality of life [5]. Pain relief and improved joint function are the main goals of OA treatment since there are no interventions available to repair the degenerated cartilage or even to slow the disease’s progression [6]. Non-surgical treatment of knee OA includes both drug and physical therapy. 

Kinesio taping (KT) has become a popular modality in physiotherapy for treating osteoarthritis in the knee joint; the proposed effects of KT include reduction in pain, enhancement of joint stability, improvement of muscle strength, and myofascial correction, in addition to functional and proprioceptive facilitation [7,8]. There is still debate surrounding the application of KT to the treatment of knee joint osteoarthritis, and the findings of systematic reviews on the subject should still be interpreted cautiously. While several systematic reviews and meta-analyses [5,9,10] concluded that KT is a safe therapeutic tool to relieve pain, enhance function, improve the isokinetic quadriceps torque, and reduce the need for painkillers in subjects diagnosed with knee osteoarthritis, others disagreed with this conclusion [11,12,13]. This highlights the need for well-designed, appropriately powered studies to completely comprehend the short and long-term effects of kinesotaping in knee osteoarthritis. 

Jenny McConnell developed the rigid nonelastic tape technique in 1984, which is still in use today, to treat patellar malalignment in patients with patellofemoral osteoarthritis (PFOA). When studying the effects of the McConnell and KT on anterior knee pain during stair ambulation in patients with patellofemoral pain syndrome (PFPS), it was found that although both the McConnell and KT were effective in reducing knee pain, the McConnell tape was found to have a superior impact on pain relief during stair ambulation [14]. Although rigid tape was initially developed to treat PFOA, it was also successfully used to treat general knee OA, improving pain, function, and overall disability [15,16]. 

A novel combination taping technique was used to overcome the limitations of individual taping techniques. It is thought that temporary biomechanical effects of rigid or non-elastic taping, which aid in realigning or offloading the joint surrounding structures, occur only while the tape is in situ. On the other hand, the underlying processes that explain the therapeutic benefits of elastic taping remain hypothesized [17]. According to a recent study, combination taping was as equally effective as ankle foot orthosis (AFO) in improving the gait parameters of children suffering from spastic diplegia when used in conjunction with a traditional physical therapy program [18]. Research on combination taping techniques’ impact on quadricep isokinetic torque, functional capacity, and disability in patients with osteoarthritis (OA) is, however, limited. Thus, the purpose of the current study was to investigate combination taping effects on patients’ osteoarthritic knee functional status, disability, and quadricep isokinetic torque.

## 2. Materials and Methods

### 2.1. Study Design and Settings

A single-blind randomized controlled trial was conducted in multiple outpatient centers in KSA. Based on the ethical rules of the Declaration of Helsinki, the Institutional Review Board at Princess Nourah bin Abdulrahman University approved the study proposal (RB Log Number: 23-0234). The study was also registered on 23 February 2023 under the trial ID NCT05755971 on clinicaltrials.gov.

### 2.2. Participants

Initially, 64 patients diagnosed by a physician with knee osteoarthritis were screened for eligibility. Participants in this study were recruited from various rehabilitation and physical therapy facilities located in the Saudi Arabian city of Jeddah. After obtaining the requested permissions, patients’ registers were reviewed and each nominated person was contacted individually by phone or in-person to obtain initial consent to participate in the study and set up screening dates. Study exclusion criteria were bilateral knee OA, severe osteoarthritis (radiologically diagnosed as grade 4), Class 2 or 3 obesity (body mass index > 35), tape allergy, symptoms suggesting another cause of knee pain, rheumatoid arthritis, steroid injection, recent knee surgery, fragile skin, or open wounds around the knee. Only 54 patients were recruited (16 men and 38 women) with the following inclusion criteria: unilateral knee OA according to the American College of Rheumatology [19], grade II or grade III knee OA as indicated by the Kellgren and Lawrence OA severity grading scale [20], and patellofemoral joint space narrowing.

An independent researcher randomly allocated eligible participants into intervention and control groups. The intervention group received a combination taping technique, which involves the application of kinesiology tape and rigid tape in conjunction with conventional physical therapy, while the control group received only conventional physical therapy. Block randomization was utilized to assign participants to groups based on gender stratification using a computer-generated random number table. Following the randomization, 28 patients (8 males and 20 females) were allocated to the intervention group, while 26 participants (8 males and 18 females) were assigned to the control group. However, three study participants dropped out, one from the control group and two from the intervention group. The study aim, procedures, and hazards were fully explained to all participants, and then they signed a written informed consent.

### 2.3. Sample Size Calculation

Considering disability as the primary study outcome, the post hoc Sample size was calculated using G*Power 3.1 (Universities, Dusseldorf, Germany) software size with an alpha of 0.05, a power of 80%, and an effect size (0.8). A priori analysis for the required sample size indicated that at least 23 subjects would be needed in each group. It is necessary to recruit a minimum of seven extra study participants to cover the maximum 15% of dropouts that may occur during the intervention and follow-up periods without compromising the study’s validity [21].

### 2.4. Outcome Measures

Independent assessors who were not aware of this study’s aim or procedures evaluated the outcome measures at baseline, after six weeks (post-intervention), and after twelve weeks from the start of the intervention (follow-up). The order in which the outcomes were measured during the post-intervention and follow-up assessments was random in order to avoid the impact of cumulative fatigue on the person’s physical performance during the subsequent test.

#### 2.4.1. Primary Outcome

Western Ontario and McMaster (WOMAC) osteoarthritis index.

WOMAC osteoarthritis index is a valid and reliable tool that is commonly used for the assessment of disability in patients with knee OA [22]. With five, two, and seventeen questions correspondingly, the index is composed of three primary domains: pain, joint stiffness, and physical activities. The index Likert version is ranked on an ordinal scale from zero to four. When it comes to knee function, a high score denotes poor status, while a low score denotes good status. The three domain scores are added together to determine the index total score [23]. The psychometric evaluation of the WOMAC Arabic version was previously reported in the literature [24].

#### 2.4.2. Secondary Outcomes

##### Six-Minute Walk Test (6 MWT) 

It has been reported that the 6 MWT is a valid and reliable method for assessing aerobic capacity [25]. The test was performed based on the guidelines provided by the American Thoracic Society [26]. A physiotherapist encouraged the subjects as they walked for 6 min at their own pace in a straight, level hallway with a hard surface, and the covered distance in meters was recorded. A greater distance denotes a higher aerobic capacity [27,28]. 

##### Timed up and Go (TUG) Test

During the TUG test, patients were required to get up from their chairs, move three meters, turn around, and then move back to the chair and take a seat. They should complete all test procedures at their own pace, not at an unsafe speed. The test duration in seconds is recorded using a stopwatch. An older adult who completes the TUG in more than 13 s is at risk of falling [29]. A recent systematic review showed that TUG is reliable and clinically valid in different populations with and without disabilities [30].

##### Isokinetic Quadricep Torque

Concentric and eccentric quadriceps peak torques were measured at an angular velocity of 60°/s at a range from 90 to 15 degrees of knee flexion using the Biodex multi-joint testing and rehabilitation dynamometer (Biodex Medical System, Shirley, NY, USA). To avoid inaccurate interpretation of the recorded data, torque measurements in the outer range were excluded [31]. The inter-subject variability caused by variations in bodyweight was then reduced by normalizing peak torque to participants’ bodyweight. The chosen velocity for isokinetic measurement is regarded as the most functional velocity that does not exacerbate pain [32].

### 2.5. Interventions

Both the intervention and control groups received a conventional physical therapy program that included the use of electrophysical agents in addition to a program of muscle-strengthening and stretching exercises. Physical agents applied were hot packs (20 min), ultrasound (5 min) using a 1.5 watt/cm intensity with a 1 MHz ultrasound head, and conventional TENS (20 min) [33]. 

The exercises consisted of stretching the hamstrings, quadriceps, and hip adductor muscles for stretching 30 s for 5 repetitions. In addition, the following strengthening exercises were included: isometric quad contractions, straight leg raises, side lying hip clams, seated leg presses, standing terminal knee extensions, and partial squats. All exercises were repeated 10 times for only one set, five days per week. The previous literature provides a thorough description of these exercises [34,35]. For Six weeks, conventional physical therapy was offered twice per week (twelve sessions).

Besides conventional therapy, the intervention group received combination taping during the first 6 weeks. Before applying McConnell and the elastic KT, the patients had their skin cleaned and shaved. To protect against allergic reactions, a mefix under-tape was first applied to cover the patella. The rigid tape was then applied to the patella in a manner that provided medial glide in addition to either medial tilt or rotation forces [36]. For fat pad unloading, another piece of tape was placed beneath the inferior pole of the patella [37].

**Figure 1:**
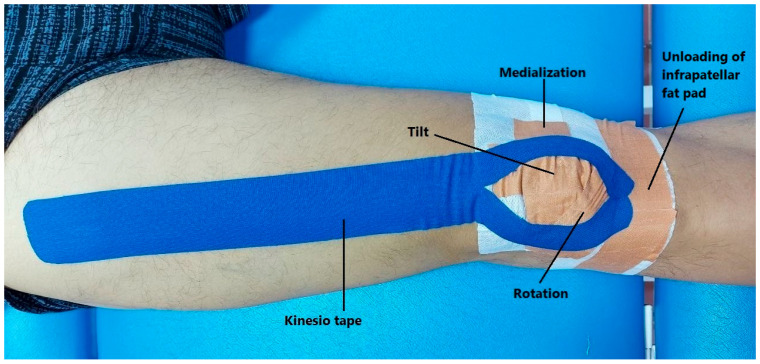
The complete application of the combination taping.

The KT was then applied while the patient was in a supine position with their hips flexed 30 degrees and their knees flexed 60 degrees. An inverted Y-shaped piece of tape was used to apply the KT facilitation technique to the quadriceps muscle. The taping started 10 cm below the anterior superior iliac spine, extended distally to the midpoint of the superior patella, and finally encircled around the patella, ending at its inferior pole. The tape was stretched to 35–50% of its original length, with the first and last five centimeters left unstretched [38]. The complete application of the combination taping is shown in Figure 1.

Twice a week, the combination tape was removed at the beginning of the physiotherapy session and then reapplied before the exercise program began. All taping procedures were performed by certified physical therapists who are well-trained in various taping techniques. The tape application took place over six weeks.

### 2.6. Statistical Analysis

Statistical analysis was conducted using the IBM Statistical Package for Social Studies (SPSS) for Windows version 25. Data were first screened for normality assumptions using Levene’s and Shapiro–Wilk’s normality tests. Moreover, skewness, kurtosis, and the presence of extreme scores were checked. Once the data were found not to violate the normality assumptions, parametric analysis was used. The Chi-square test and unpaired *t*-test were used for comparisons of the demographic data of the intervention and control groups. Meanwhile, repeated measures Multivariate Analysis of Variance (MANOVA) was used to differentiate the means of both groups for all the measured outcomes. Then, Tukey’s post hoc test was performed in cases of significant results. An alpha level of 0.05 was chosen as the significance level.

## 3. Results

**Figure 2:**
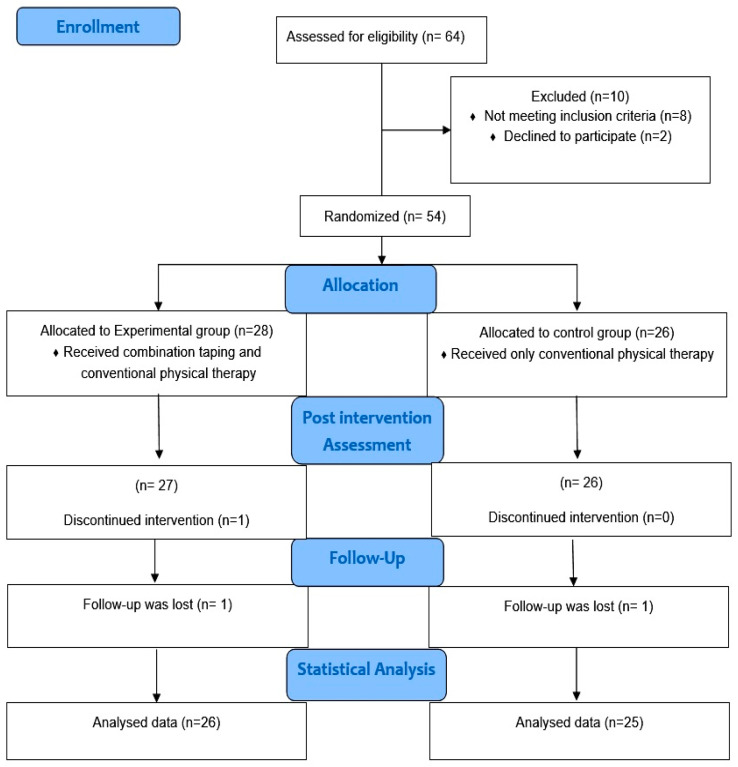
Flowchart of study procedures.

This study was conducted starting in April, and data collection ended in November 2023 and included 54 participants. Three study participants dropped out, one from the control group and two from the intervention group, as shown in the flowchart of the study procedures (Figure 2). 

Table 1 displays the demographic and clinical data of both groups. The means of both groups in terms of age, gender, weight, and BMI were not statistically different based on unpaired *t*-test analyses; also, the distribution of gender, the onset of symptoms, and the OA severity grade were not statistically different according to the Chi-square test.

### 3.1. Within-Group Comparisons

#### 3.1.1. The Intervention Group

According to MANOVA, the post-intervention means for the WOMAC index, 6 MWT, concentric, and eccentric quadricep torques were all significantly higher than the baseline means (*p* = 0.001, 0.002, 0.001, and 0.001, respectively), while the post-intervention meantime for the up and go test was significantly lower than the baseline value (*p* = 0.001). Furthermore, for the WOMAC index and the six-minute walk test, the means at the follow-up assessment were significantly higher than those at the post-intervention (*p* = 0.001, 0.016, respectively) and the mean time for the up and go test was significantly lower than that of the post-intervention assessment (*p* = 0.001). However, the means of the concentric and eccentric quadricep torques did not significantly differ between the post-intervention and follow-up assessments. (*p* = 0.568 and 0.391, respectively) 

#### 3.1.2. The Control Group

The MANOVA analysis revealed that the means for the WOMAC index and six-minute walk test were significantly higher than the baseline means (*p* = 0.003 and 0.002, respectively), while the post-intervention time for the up and go test was significantly lower than the baseline value (*p* = 0.004). On the other hand, the means of concentric and eccentric quadricep torques did not differ between baseline and post-intervention measurements (*p* = 0.154, 0.116, respectively). In the same context, there were no significant differences between the post-intervention and follow-up assessment means for all outcome measures (*p*-value > 0.05). All within-group comparisons are shown in Table 2.

### 3.2. Between-Group Comparisons

Overall, with respect to the outcomes assessed, the interaction effect (time × group) was statistically significant (F = 62.47, *p* = 0.001). 

**Figure 3:**
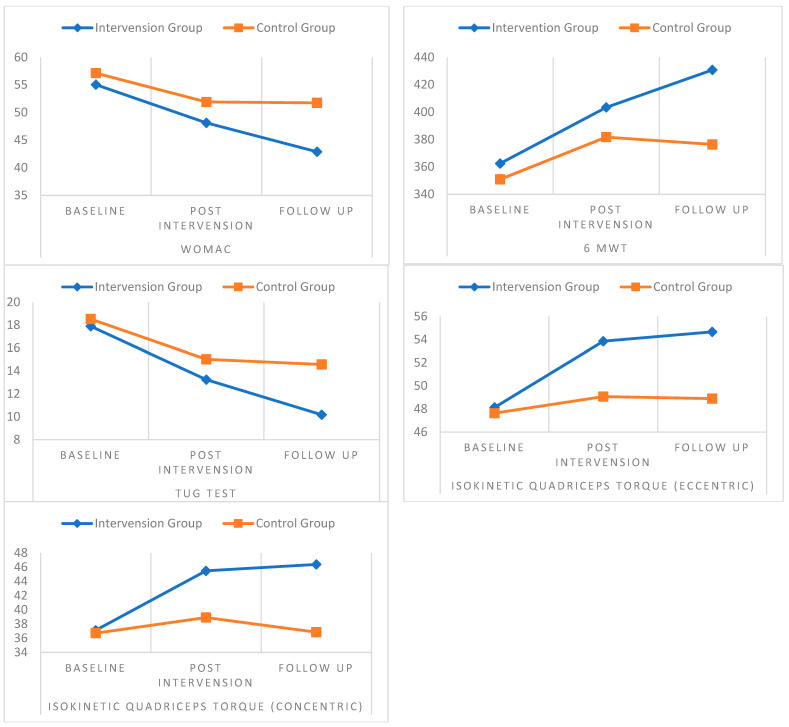
Outcome measures during post-intervention and follow-up assessments.

The pre-intervention means of all the outcome measures for the intervention and control groups were not significantly different (*p*-value > 0.05). However, the intervention group’s means for the WOMAC index, six-minute walk test, and concentric and eccentric quadricep torques were all significantly higher than those of the control group post-intervention assessment (*p*-value = 0.35, 0.39, 0.0002, and 0.0005, respectively) with higher significance level during follow-up assessment (*p*-value = 0.0001 for all measures). Moreover, the intervention group’s meantime for the up and go test was significantly lower than those of the control group during post-intervention and follow-up assessments (*p*-value = 0.0005 and 0.0001, respectively). All between-group comparisons and effect sizes are shown in Table 2. Additionally, Figure 3 summarizes the progress of all outcome measures during post-intervention and follow-up assessments. 

## 4. Discussion

This is the first study, as far as we know, to investigate the impact of the combination taping technique on patients with knee OA regarding their functional status, degree of disability, and quadricep isokinetic torque. When combination taping was added to conventional physical therapy, the WOMAC index, the six-minute walk test, and the timed up and go test all showed improvements in knee function and disability over the short and long term. These results were significantly superior to those attained with conventional physical therapy alone. Furthermore, compared to the control group, the combination taping group showed improvement in both the concentric and eccentric quadricep torque.

The improvements in knee joint disability, as determined by the WOMAC index in this study, can be mainly attributed to the beneficial effects of both types of taping on pain perception. Biomechanical faults due to patellar maltracking are believed to contribute to the production of knee joint symptoms. From a mechanical perspective, McConnell [37] explained that rigid taping corrects the misaligned patellar position and repositioned the bone inside the trochlea groove. Better load distribution and surface contact would lead to reduced pressure for the same magnitude of applied forces at the patellofemoral joint. Moreover, offloading the fat pad might ease the pressure on this frequently irritated soft tissue. These findings are consistent with earlier research. NG and Cheng [39] found that rigid taping reduced pain by nearly 50% according to the VAS score in subjects with patellofemoral pain. Also, Mackay et al. observed that during three testing conditions—a self-selected pain provocative task, running, and a single-leg squat—Mulligan knee taping decreased pain and enhanced lower limb biomechanics in female patients with patellofemoral pain (PFP) [40]. Similar findings regarding rigid taping’s efficacy in PFP were published by Hickey et al. [41] and more recently by Yoon and Son [14].

Regarding the Kinesio tape, it was reported that the tape elasticity and the applied tension improve lymphatic and vascular flow as well as skin mobility during motion, and this process directly affects how pain is perceived [38]. Activation of cutaneous mechanoreceptors with consequent neurological suppression is another factor that may contribute to pain relief [42]. This could alter how proprioception and cutaneous mechanoreceptors interact, reducing the sensation of stiffness [43]. These results support the findings of a meta-analysis by Lu et al. [5], who found that taping can help patients with knee OA experience less pain and joint dysfunction. On the other hand, the outcomes do not align with the findings of Wageck et al. [44], who concluded that, in comparison to sham tape, KT did not improve knee joint disability in older adults with osteoarthritis, as determined by the WOMAC index after four days of tape application. This could be explained by the short duration for which the tape was applied and the variations in the taping techniques used in the two studies. 

The experimental group’s improved disability may also be explained by the conventional physical therapy that was given as an adjuvant. Knoop et al. [45] reported that the WOMAC index was improved by about 14% in subjects with knee osteoarthritis after a 12-week knee stabilization protocol. According to Hmamouch et al. [46], a difference in WOMAC score must be greater than 12% of the initial measurement value to be considered clinically significant in patients with OA. Therefore, it is possible to view the WOMAC changes in the present study as having strong clinical relevance with an improvement exceeding 20% of the baseline value.

The improvement in aerobic capacity and functional status, as measured by a 6 MWT and TUG test, could be explained by changes in pain perception, joint disability, proprioceptive acuity, quadricep strength, neuromotor control, and knee range of motion. Callaghan et al. [47] reported an improvement in knee joint proprioception, as measured by active and passive angle reproduction on an isokinetic dynamometer after patellar taping. In the same context, Hanafy and Abdallah [16] found that patellar Mulligan rigid taping significantly reduced stair climbing time and increased 6 min walking distance in patients with OA of the knee joint when compared to sham taping or no taping. 

Furthermore, using kinesio tape has been shown in multiple studies to significantly increase knee flexion range [33,48,49]. These results are in disagreement with those of Rahlf et al. [2], who showed no difference in the timed 10 m test after the application of kinesio tape on the knee joint for three successive days. The reasons for these discrepancies in results could be explained by the brief duration of the tape application and the type of functional waking test chosen in Rahlf’s study. The extremely short distance covered during the 10 m walk test raises questions about its suitability for detecting improvements in the ambulation capacities of individuals with the diagnosis of osteoarthritis.

Finally, the improvement in quadricep peak torque in the experimental group may be attributed to the way the patellar realignment changed the leverage and mechanical advantage the quadricep muscles received. A greater mechanical advantage means that less force is required to produce the same torque, and fewer compressive forces are generated in the patellofemoral joint [50]. Additionally, patellar realignment maximizes the vastus medialis oblique (VMO) muscle’s length–tension relationship, a crucial stabilizing element of the muscle that promotes a more powerful and effective contraction of the entire quadriceps [51]. A prior study found that when patellar malalignment was corrected with rigid taping in patients with knee OA, there were instant improvements in quadricep peak torque [16].

Another explanation is that tapping increases peak torque in the quadricep muscle by lowering pain-induced inhibition of the muscle. It has long been known that pain in the knee joint causes reflex inhibition, which in turn contributes to quadricep weakness [52]. A final explanation is that taping may have raised the peak torques of the quadriceps muscles by activating sensory receptors and generating proprioceptive impulses, which enhance muscle function [8]. 

The effectiveness of taping for strengthening quadriceps, however, is a topic of much debate in the literature. Numerous studies revealed that applying tape did not significantly affect muscle strength, which disagrees with the results of our study [33,53,54]. There are numerous perspectives from which to view this glaring contradiction. The first is the different approach to taping, since combination taping has not been used in any of the earlier research. Furthermore, since many of these studies focus on the immediate effect rather than the short- or long-term effects, there is a difference in the timing of the post-taping assessment. In these studies, the objective isokinetic dynamometer was substituted with other instruments for assessing strength. Lastly, it is unclear if the tape was worn or taken off during the post-intervention assessment. Positive results are reported when the tape is applied during the assessment, which was the case in this study and the study by Anandkumar et al. [8], whose findings are consistent with ours. This also might explain why a non-significate difference was found between the post-intervention and follow-up assessments in our study as follow-up assessment was performed with the tape removed. 

It is important to address this study’s limitations, however. First, the study groups’ outcomes were not compared with those of individual taping techniques or with sham taping, which applies no tension. Second, while the literature reported on several elastic taping techniques, this study only adopted the quadriceps facilitatory technique. Third, the daily physical activity levels of the study participants could not be standardized, which could have an impact on the outcome measures. Finally, although the involved physical therapists were given a clear and comprehensive description of the conventional physical therapy program, this study is being conducted across multiple centers, making it impossible to control the quality of the therapy provided. 

## 5. Conclusions

The current study’s findings showed that, in the short term, patients with unilateral knee OA who received novel combination taping in addition to conventional physiotherapy had better improvements in disability, function, and quadricep torque than those who received conventional physiotherapy alone. Except for quadricep isokinetic torque, outcome measures improved even more in the combination taping group after six weeks of follow-up, but not in the control group. Future studies comparing individual taping techniques with the recently studied combination taping are strongly recommended.

## Figures and Tables

**Table 1:** The demographic and clinical data of both groups.

	Intervention Group *n* = 26 Mean (SD)	Control Group *n* = 25 Mean (SD)	*p*-Value
Age (years) ^a^	67.46 (8.55)	66.31 (7.67)	0.423
Height (m) ^a^	1.72(0.15)	1.69 (0.17)	0.506
Weight (kg) ^a^	91.63 (6.37)	90.85 (7.8)	0.696
BMI (kg/m^2^) ^a^	30.82 (2.74)	31.61 (1.2)	0.191
Men/women ^b^	8/18	8/17	
The onset of symptoms (years) ^b^	6.8	7.2	
OA grade, II/III ^b^	9/17	7/18	

Data are illustrated as mean (standard deviation); ^a^ refers to unpaired *t*-test; ^b^ refers to Chi-square test.

**Table 2:** Within and between-group comparisons for all outcome measures.

Variables	Groups	Baseline	Post Intervention	Follow Up	Pre- vs. Post-Intervention Comparison		Post-Intervention vs. Follow-Up Comparison	
		Mean (SD)	Mean (SD)	Mean (SD)	*p*-Value	MD (95% CI)	*p*-Value	MD (95% CI)
WOMAC	Intervention group	55.04 (4.73)	48.13 (5.96)	42.91 (5.16)	0.001	−6.91 (−9.8–−3.81)	0.001	−5.22 (−8.32–−2.11)
	Control group	57.14 (3.68)	51.91 (6.52)	51.76 (5.64)	0.001	−5.23 (−8.24–−2.21)	0.889	−0.24 (−3.7–3.21)
	*p* value *	0.083	0.035	0.0001				
	Effect size				0.58		1.41	
6 MWT (m)	Intervention group	362.52 (39.65)	403.42 (33.2)	430.74 (45.1)	0.002	40.9 (20.52–1.27)	0.016	27.32 (5.23–49.40)
	Control group	350.92 (41.32)	381.72 (39.7)	376.36 (32.87)	0.03	30.8 (7.75–53.84)	0.605	−2.36 (−26.08–15.36)
	*p* value *	0.284	0.039	0.0001				
	Effect size(Cohen’s d)				0.59		1.37	
TUG test (s)	Intervention group	17.89 (2.14)	13.24 (1.58)	10.17 (1.42)	0.004	−4.65 (−5.69–−3.60)	0.001	−3.07 (−3.90–−2.23)
	Control group	18.52 (2.4)	15.01 (1.82	14.56 (2.16)	0.001	−3.51 (−4.70–−2.31)	0.429	−0.25 (−1.58–0.68)
	*p* value *	0.326	0.0005	0.0001				
	Effect size				0.89		1.65	
Isokinetic quadriceps torque (Nm/Kg) Concentric	Intervention group	37.12 (5.29)	45.48 (6.27)	46.39 (5.10)	0.001	8.36 (5.12–11.59)	0.568	1.06 (−4.09–2.27)
	Control group	36.72 (4.77	38.92 (5.46)	36.85 (4.62)	0.154	2.2 (−4.94–0.80)	0.163	−1.07 (−5.62–1.25)
	*p* value *	0.778	0.0002	0.0001				
	Effect size				0.94		1.72	
Isokinetic quadriceps torque (Nm/Kg) Eccentric	Intervention group	48.13 (2.71)	53.85 (3.31)	54.66 (3.45)	0.001	6.72 (4.03–7.40)	0.391	0.21 (−2.69–1.07)
	Control group	47.63 (2.69)	49.07 (3.52)	48.89 (3.02)	0.116	1.44 (−0.34–3.22)	0.847	−0.08 (−1.68–2.04)
	*p* value *	0.511	0.0001	0.0001				
	Effect size				0.98		1.86	

Data are illustrated as mean (SD) standard deviation. WOMAC—Western Ontario and McMaster Universities Osteoarthritis Index, 6 MWT—six-minute walk test, TUG—timed up and go, m—meter, s—second, Nm/Kg—newton meter/kilogram, CI—confidence interval. *p*-value is within-group significant at <0.05. *p* value * is between the group’s significance level.

## Data Availability

Data are available upon reasonable request.
